# An Easy Method to Determine the Effective Conductivity of Carbon Fiber Composites Using a Wall Perturbation Approach

**DOI:** 10.3390/ma16062404

**Published:** 2023-03-17

**Authors:** Moritz Engler, Guido Link, John Jelonnek

**Affiliations:** Institute for Pulsed Power and Microwave Technology (IHM), Karlsruhe Institute of Technology (KIT), 76131 Karlsruhe, Germany

**Keywords:** carbon fiber reinforced plastic, conductivity measurement, perturbation method

## Abstract

Carbon-fiber-reinforced plastics (CFRPs) are of increasing popularity in a wide range of applications, and microwave curing promises significant reduction in processing times. However, for the design of an efficient microwave curing system, the composites’ effective material parameters must be known. This work presents a measurement system using a wall perturbation approach with a coaxial cavity to determine the effective conductivity of a CFRP along the fiber direction.

## 1. Introduction

Carbon-fiber-reinforced plastic composites are increasing in popularity. Due to superior strength to weight ratio compared to steel and aluminum and high corrosion resistance, CFRP composites are the candidates of choice for high-performance applications as required for, e.g., the aerospace and automotive industries. The major drawback that prevents their widespread use is the relatively high cost due to the complex manufacturing process. The majority of CFRPs are produced in batch processes using classic autoclaves for the curing of the resin. To achieve an even cure of the resin matrix in a classic autoclave, the heating rate must be sufficiently low to allow homogeneous heating of the component, which increases the cycle time, especially for large components. One possible method of improving this process is to substitute or supplement the conventional heating with microwaves. Due to the volumetric nature of microwave heating, a homogeneous temperature of the work piece can be achieved significantly faster. Additionally, using microwave heating offers significantly more energy-efficient processes. However, proper design of a microwave oven is difficult, particularly as the conducting carbon fibers that are enclosed within the isolating resin matrix create a material with highly anisotropic behavior. This is particularly valid in the case of a composite with unidirectional fiber reinforcement. While there is a long history of theoretical considerations on the effective electric parameters of fiber materials, there are only few sources which provide some values of the effective electrical parameters of CFRPs [1,2,3,4]. Those values are not consistent and differ from each other significantly, which is particularly true for cases for which the electric field is parallel to the reinforcing fibers. Considering the theoretical models behind the values, some describe the material in this polarization as a dielectric with high permittivity and losses, while others describe it as a lossy conductor.

Zhang et al. reported a permittivity of approximately $\varepsilon_{r}^{\prime }=125$ for a cured CFRP sample with parallel polarization [2], which is similar to the findings of Zhou et al., who measured a permittivity of $\varepsilon_{r}^{\prime }=200$ [3]. In contrast to that, Jaleel Akhtar et al. reported values of $\varepsilon_{r}^{\prime }=1500$ up to $\varepsilon_{r}^{\prime }=4500$ [4]. Moreover, while Zhang et al. and Zhou et al. observed a significant change in effective material properties during cure for both polarizations [2,3], the measurements of Zhou et al. in [5] show nearly identical reflectivity transmittance or absorptivity between cured and uncured samples for the case of orthogonal polarization. This illustrates that effective parameters of CFRPs always need to be evaluated for the specific material. This paper concentrates on a measurements method for the characterization of CFRPs for which the electric field is parallelly polarized. It considers that the fiber volume content has a large influence on the effective permittivity of the CFRP composite. The fiber volume content is defined as the ratio between the overall fiber volume within the sample and the sample’s total volume.

Mixing rules for the prediction of effective parameters of fibrous materials have a long tradition [6]. The most common method used for the prediction of the effective permittivity $\varepsilon_{eff}$, of CFRPs is based on the generalized form of the Maxwell–Garnett Mixing Rule for ellipsoidal inclusions [7]:

(1) $$\varepsilon_{eff}=\varepsilon_{1}\left(1+\frac{f_{2}\left(\varepsilon_{2}-\varepsilon_{1}\right)}{N\left(1-f_{2}\right)\left(\varepsilon_{2}-\varepsilon_{1}\right)+\varepsilon_{1}}\right)$$

with $\varepsilon_{1}$ denoting the permittivity of the background material and $\varepsilon_{2}$ denoting the permittivity of the inclusion—in this case, the carbon fiber. $f_{2}$ refers to the volume fraction of the inclusions, and N is the depolarization, factor which depends on the shape of the inclusions. For the unidirectional CFRP, the ellipsoids can be assumed to be infinitely stretched in one dimension, resulting in a wire of infinite length and infinitesimal radius. The depolarization factor for a thin wire aligned along the z-axis is given as:

(2) $$N=\left[\begin{array}{ccc} 1/2 & 0 & 0\\ 0 & 1/2 & 0\\ 0 & 0 & 0 \end{array}\right]$$

Hence, Equation (1) reduces to:

(3a) $$\varepsilon_{⊥}=\varepsilon_{1}+2f_{2}\varepsilon_{1}\frac{\varepsilon_{2}-\varepsilon_{1}}{\varepsilon_{1}+\varepsilon_{2}-f_{2}\left(\varepsilon_{2}-\varepsilon_{1}\right)}$$

(3b) $$\varepsilon_{\|}=f_{1}\varepsilon_{1}+f_{2}\varepsilon_{2}$$

where $\varepsilon_{⊥}$ and $\varepsilon_{\|}$ are the effective permittivities for the cases of orthogonal and perpendicular carbon fibers with respect to the electric field and $f_{1}=\left(1-f_{2}\right)$ is the volume content of the background material. As the permittivity of a good conductor is not defined, the imaginary part of the complex permittivity of the carbon fibers is calculated from its conductivity, while the real part is assumed to be one. While these mixing rules are often used to describe carbon fiber composites, their derivation assumes a low volume percentage of inclusions, while fiber composites often possess high fiber volume contents. The results of the mixing rule should therefore be checked via measurement before the rule is used for the design of a microwave system.

Due to the high conductivity of the carbon fibers, the effective permittivity of CFRP composites is significantly higher than those of most other materials. It requires very thin samples in transmission reflection methods for effective parameter determination [4]. Precise placement of those thin samples inside the waveguide is challenging and prone to placement errors. Small air gaps between the sample and the waveguide walls or variations in sample thickness can introduce significant errors, especially for materials with high permittivity and losses [4].

The fiber conductivity $\sigma_{cf}$ is in the order of $6\cdot 10^{4}S/m$, while the imaginary part of the dielectric constant for the matrix material is several orders of magnitude lower. Therefore, the loss term introduced by the matrix material can be neglected and the effective conductivity $\sigma_{eff}$ of the composite is determined by the carbon fiber conductivity and the fiber volume content:

(4) $$\sigma_{eff}=f_{2}\sigma_{cf}$$

Due to the high effective conductivity, the CFRP can be considered to behave like a lossy conductor. To measure the effective conductivity of the CFRP, a wall perturbation approach is chosen. Such resonant perturbation methods, e.g., the wall perturbation method is based on the assumption that the electromagnetic field and surface currents remain nearly constant if a sample is introduced in the empty cavity. Therefore, an observed change in resonant frequency and quality factor can be directly related to the material parameters of the sample. For the wall perturbation method, it is assumed that the perturbed and unperturbed state differs only in the surface impedance $\Delta Z_{S}$, which leads to the following Equation (8):

(5) $$\Delta \omega =\left(j\int_{S}^{}H_{1}\cdot H_{1}\Delta Z_{s}dS\right)/4W.$$

Here, Δω is the change in the complex resonant frequency of the lossy cavity. $H_{1}$ is the magnetic field inside the unperturbed cavity, S is the surface of the cavity, and W the energy stored inside the cavity. For practical applications, the equation can be simplified further:

(6) $$\Delta f=\frac{j}{2\pi }\Gamma \Delta Z_{s}.$$

Here, Γ is a constant containing the unknown quantities of Equation (5). Its value is determined by the shape and materials of the resonator [8]. The value is found by measuring two samples with known surface impedance. As the surface impedance considers losses, the shift in the complex resonance frequency Δf is also a complex quantity, where the real part corresponds to the measured frequency shift and the imaginary part corresponds to the shift of the inverse of the quality factor. Therefore, the surface resistance of the sample $R_{s2}$ can be calculated from:

(7) $$R_{s2}=R_{s1}+A\left(\frac{1}{Q_{2}}-\frac{1}{Q_{1}}\right)$$

$R_{s1}$ is the surface impedance of the original cavity wall, $Q_{1}$ and $Q_{2}$ are the quality factors of the empty cavity and the perturbed cavity, respectively. A is a resonator constant. The surface resistance relates to the sample conductivity by:

(8) $$R_{s}=\sqrt{\omega \mu_{0}/2\sigma }.$$

The sample conductivity can be found by inserting Equation (8) into (7) and solving for σ. $R_{s1}$ can be calculated from the known conductivity of the cavity walls, and A can be found by comparing the unperturbed Q-value of the cavity to that of a sample of known conductivity.

## 2. Materials and Methods

### 2.1. Measuremet Set-Up

Due to the axially anisotropic nature of the CFRP, the effective conductivity can only be measured if the surface currents on the sample are aligned with the carbon fibers. The following is from Maxwell’s equations for the surface currents at a conducting boundary [9]:

(9) $$J_{s}=n\times H$$

To achieve a surface current whose direction is parallel to the carbon fibers, a resonator based on a transverse magnetic (TM) or transverse electromagnetic (TEM) waveguide can be used. For a straight waveguide with constant cross section this results in parallel surface currents that are in line with the propagation direction of the modes. To avoid issues with unknown contact resistances between the cavity and the sample, the border of the wall section that is replaced by the sample should coincide with points where the surface currents vanish. For a straight cavity, lines with zero surface currents transverse to the propagation direction of the modes exist due to the equality of forward and backward propagating modes in the cavity. Here, a straight waveguide with rectangular cross section is used. Assuming $H_{z}=0$ the Equation (9) leads to $J_{s}=\pm H_{y}z$. In addition, the normal magnetic field is zero.

(10) n·B=0

This leads to $H_{x}=0$ as long as the resonator is not filled with a medium for which the permeability is larger than one. In the same way, a horizontal wall leads to $J_{s}=\pm H_{x}z$ and $H_{y}=0$. In the waveguide corner, where the horizontal and vertical walls meet, the conditions for both walls must be fulfilled. Hence, the magnetic field and the surface currents must vanish. This consideration leads to a coaxial cavity with rectangular outer conductor. For this coaxial cavity, operation at the second TEM resonance is preferred over the TM_112_ mode. Since the TM_112_ mode is degenerated from the TE_112_ mode, it cannot be guaranteed that there is no coupling between these modes inside the cavity, in which case unwanted surface currents at the sample edges might occur. In this paper, the cavity length l is chosen to be l = 122 mm, which results in a resonance frequency of about 2.45 GHz. 

**Figure 1:**
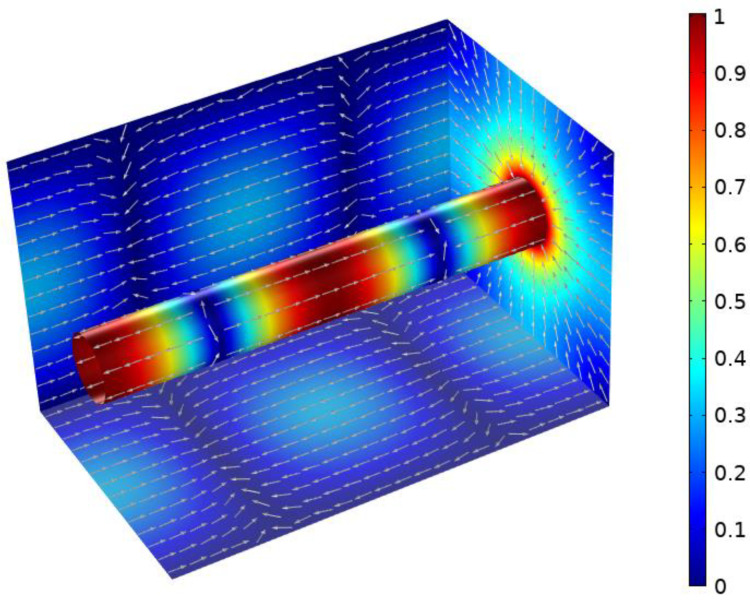
Normalized current density and direction in coaxial resonator with rectangular outer conductor.

**Figure 2:**
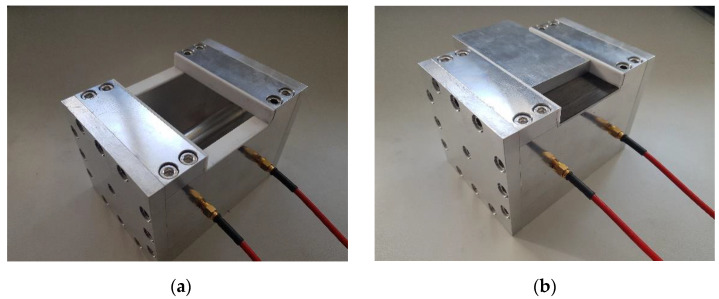
Assembled cavity with empty sample holder (**a**). Cavity with prepreg sample placed on the sample holder. The aluminum lid is partially placed on top of the sample for illustrative purposes (**b**).

Coupling is realized by using two coaxial pins placed at the sidewall at a quarter and three quarters of the cavity length. The sides are 75 mm long. This presents a good compromise between a convenient sample size and a sufficiently high quality factor. The ideal radius of the center conductor is determined by using the commercial 3D EM simulation software CST Microwave studio. For a given length of the feed pin of 3 mm, the ideal center conductor radius is found to be 9 mm. Figure 1 shows the normalized current density at 2.45 GHz for this cavity configuration. As can be seen, the side walls offer the desired parallel surface currents with a border of vanishing currents. The highest surface current density occurs along the center conductor and the transition from the side walls to the center conductor. Therefore, the electrical contact between those parts is of particular importance in achieving a high quality factor. To ensure this, knitted wire mesh gaskets are inserted between the center conductor and the end walls. The top wall of the cavity only covers one fourth of the cavity length on each side, such that a central opening is created. The edges of this opening coincide with the areas where the surface currents vanish. An aluminum lid was manufactured to fit precisely into the resulting opening. The aluminum lid serves as a reference sample. When characterizing a material, the lid can be replaced by the sample or the sample can be placed between the cavity and the lid. As imperfections during manufacture might lead to small deviations from the theoretical surface current distribution, PTFE insulation is placed at the contact surfaces between the sample and the cavity to avoid points of undefined electrical contact. The fully assembled cavity is shown in Figure 2. 

For the classical wall perturbation method, Equations (7) and (8) are used to calculate the sample conductivity. The first step is to calculate the value of the cavity constant A using a reference of well-known conductivity. In order to uphold the assumption made for the derivation of Equation (5) that the field configuration and energy stored in the cavity do not change between the perturbed and unperturbed state, the surface impedance of the reference sample and the sample under test must be similar. However, the conductivities of most metal materials suitable as a reference sample are in the order of $10^{7}$ S/m, while the expected conductivities of the CFRP samples are in the order of $10^{4}$ S/m. Therefore, the classic perturbation calibration is replaced by a full wave simulation approach using CST Microwave Studio. Doing this allows for a single reference measurement using an aluminum plate to generate the characteristic curve, which relates the measured quality factors with the sample conductivities. Since aluminum is a sufficiently good conductor, the quality factor with the aluminum sample is close to the quality factor for a perfect conducting sample. This means the sensitivity of the quality factor towards the sample conductivity is low and that uncertainties in the aluminum reference’s conductivity have little influence on the generated characteristic curve.

**Figure 3:**
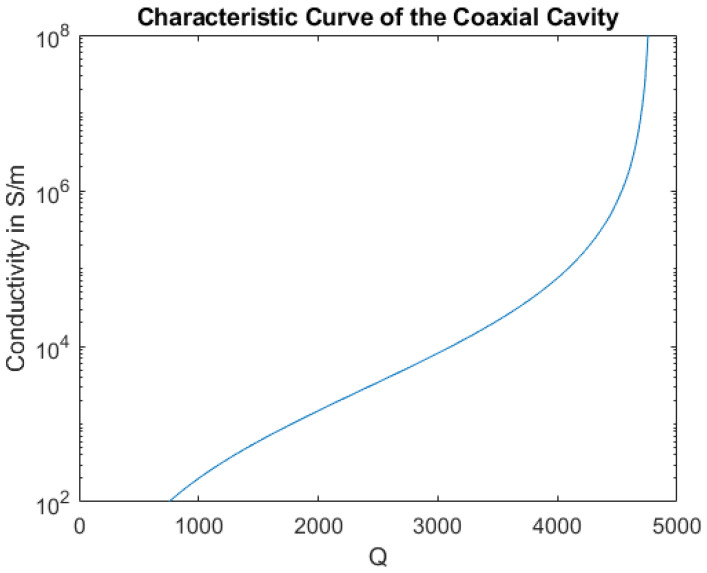
Characteristic curve of the measurement setup, calculated using CST Microwave Studio.

The first step in generating the characteristic curve is to measure the unperturbed cavity covered only by the aluminum lid as a reference. The measurement of the quality factor for the aluminum lid gave mean values between 4855 and 4856 for repeated measurements with varying lid orientations. The highest standard deviation over one measurement interval was 3.9. The accuracy of the reference measurements using the aluminum lid is therefore limited by the accuracy of the quality factor measurement achieved with the network analyzer. When measuring over a prolonged period of time, a slight drift can be observed in the value of the quality factor. It is therefore advisable to measure the reference before and after measurements of the samples under test. While the repeatability of the reference measurement is excellent, the absolute value of the quality factor is significantly lower than in the simulation. This is thought to result from imperfect contact between the center conductor and side walls with the endplates as well as from imperfections in the surface quality of the cavity walls. As the contributions of these additional loss mechanisms on the quality factor cannot be separated, they are combined into a single additional loss term and added to the simulation. In this case, a surface roughness was introduced and adjusted to match the results of the measurement in the CST simulation. When the simulation and the measurement of the reference are in agreement the characteristic curve of the cavity can be acquired by sweeping the sample conductivity in the CST model. The resulting curve shown in Figure 3 can then be used to relate the Q values, measured with a sample on the cavity, to the samples’ conductivity. In the range from $10^{3}$ S/m to $10^{5}$ S/m, which is the typical conductivity range for carbon fiber composites, the characteristic curve has a shallow slope, allowing very accurate measurements in this range. At high conductivities, the slope increases significantly, making the measurement setup impractical for the measurement of samples with conductivities above $10^{6}$ S/m. 

### 2.2. Materials under Test

Classical conductivity measurements cannot be used to verify the new method proposed here since the insulating matrix material covers the conducting fibers in the CFRP samples. Contacting the sample reliably is therefore impossible. However, mixing models or full wave simulations can be used to check if the measurement results are reasonable. To compare the measured effective conductivity with the mixing model, the conductivity of the reinforcing fibers had to be determined. The sizing agent of the Toray T300 carbon fibers was removed using an acetone bath over 48 h. Single carbon fibers of various lengths were fixed to adhesive tape for ease of handling. The fiber ends were then attached to copper tape and contacted using conductive silver paint. The resistance of a single fiber could then be measured using a multimeter.

CFRP samples were prepared from unidirectional prepreg and from carbon-fiber-reinforced thermoplastics (CFRTP). The prepreg samples were made by stacking four layers of prepreg, resulting in a sample thickness between 1.1 mm and 1.2 mm. The thermoplastic samples were created by aligning segments of continuously fiber-reinforced 3D printer filaments and then bonding them together in an oven at 230 °C for 10 min. The samples each consist of two layers of filaments, which results in a sample thickness of 0.5 mm to 0.8 mm. By alternating between carbon fiber reinforced filaments and conventional filaments, the fiber volume fraction of the samples can be varied. Four types of CFRTP samples were created. Samples 1 and 2 were prepared from one fiber reinforced filament alternating with two unreinforced filaments. Sample 3 consists of an alternation of one reinforced and one unreinforced filament. Samples 4 and 5 alternate between two reinforced and one unreinforced filament, and Sample 6 consists only of reinforced filaments. The carbon fiber reinforced printing filament is cylindrical, with a diameter of 0.35 mm. It is reinforced by Toray T300 1K carbon fibers. This leads to a fiber volume fraction of 39%. The unreinforced filaments are of rectangular cross section with 0.4 mm side lengths, which leads to fiber volume contents of 22%, 15%, and 9% for the samples with reduced fiber count. 

**Figure 4:**
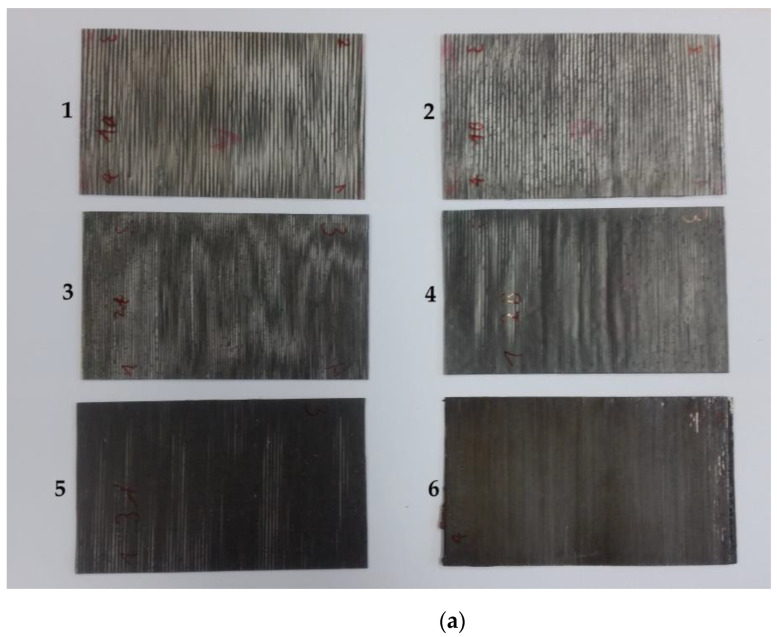

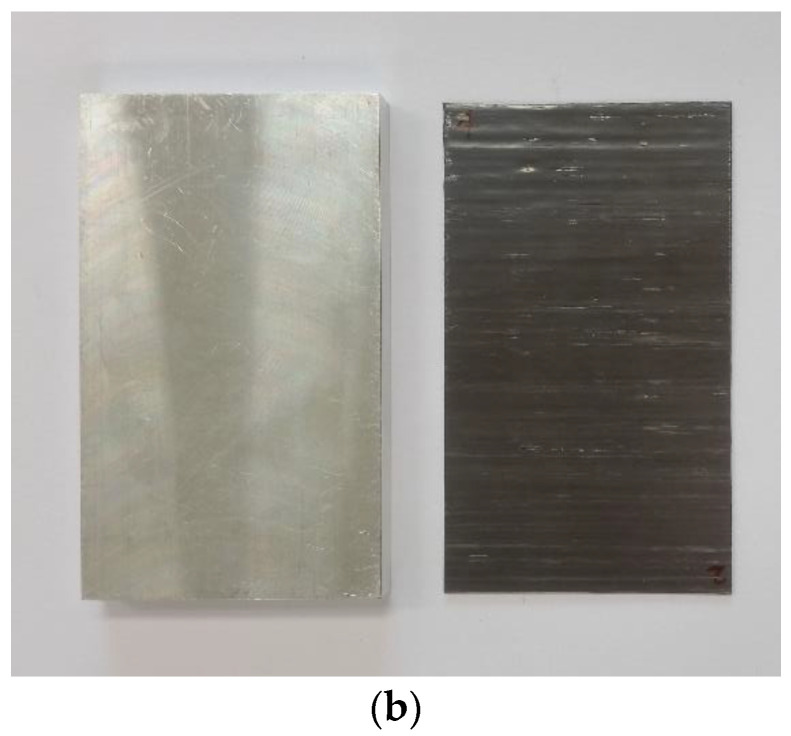
Pictures of samples under test: (**a**) CFRTP samples with different fiber volume contents. (**b**) Aluminum reference (left) and prepreg sample (right).

Pictures of samples are shown in Figure 4. For the prepreg samples in Figure 4b, only one of the samples is depicted as there is no visible difference between the individual samples.

**Figure 5:**
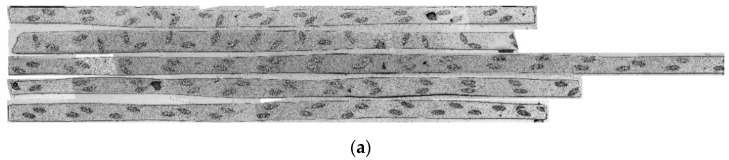

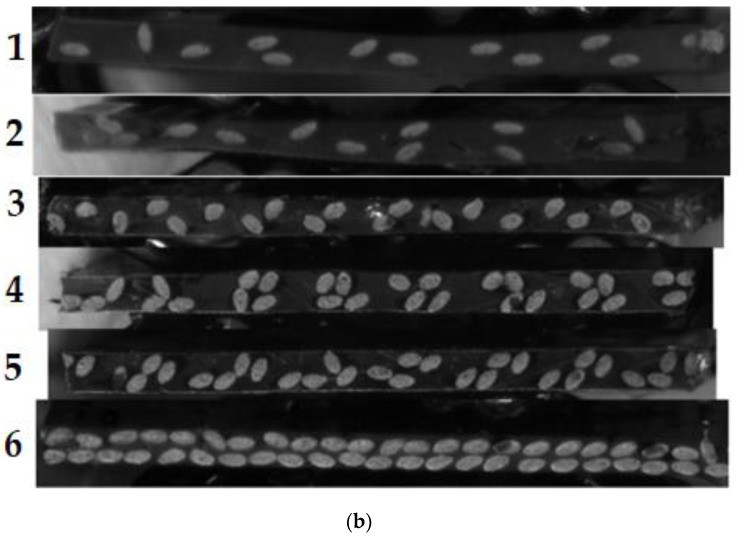
Microscope images: (**a**) composite of microscope images of sample 1. (**b**) Segments from Samples 1 to 6, beginning with Sample 1 at the top.

As is evident from Figure 4a, the distribution of carbon fibers in the CFRTP samples is not homogeneous. This is due to the manufacturing process, as the fiber bundles within the reinforced 3D printing filaments do not spread out during the fusing of the filaments. Within the reinforced filaments, the fibers are also not evenly distributed. Instead, they accumulate in the center as a compact bundle covered by a layer of pure thermoplastic. To obtain a better understanding of the sample structure, microscope images were made from segments of all six samples. For Sample 1, images were made covering the whole length of the sample to get an estimate for the variation of the fiber distribution across the sample. For the remaining samples, images were only made for a small section of each sample. The microscope images are presented in Figure 5.

**Figure 6:**
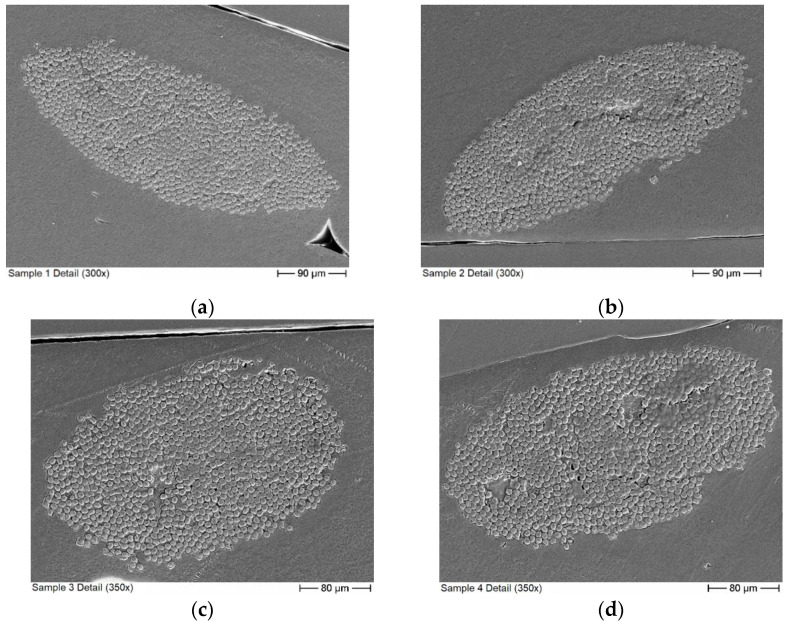

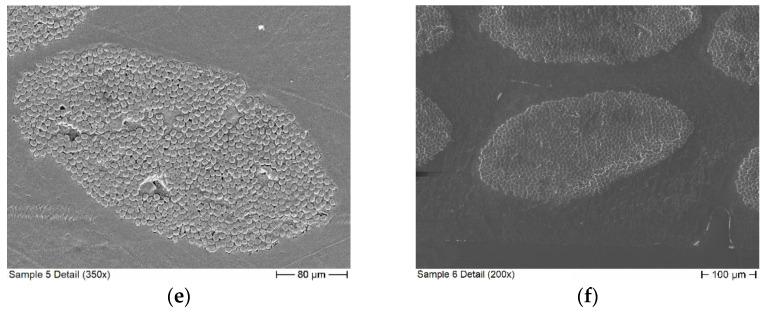
SEM images showing close-ups of fiber bundles, where (**a**–**f**) depicts a bundle from sample 1–6 respectively.

In addition to the sample pictures above, close up images of individual fiber bundles were made using scanning electron microscopy (SEM) to obtain a better understanding of the fiber distribution within the bundle. Some of these pictures are presented below in Figure 6.

### 2.3. Measurement Procedure

For a series of measurements, the first step is to measure the aluminum lid as a reference value, as mentioned before. This is necessary as the cavity’s quality factor and resonance frequency can shift slightly over consecutive days. Following the reference measurement, the sample measurement can begin. For this, the samples are first placed on the resonator and covered by the aluminum lid. As the aluminum lid is precision-fit to the sample holder, this ensures optimal sample placement. Data are collected over a 100 s interval. Afterwards, the lid is removed, and the sample is measured for another 100 s. In this way, the first measurement provides an upper limit and the second measurement a lower limit for the sample conductivity. As a next step, the sample will be rotated to a different orientation and measured again with and without the lid. This procedure is repeated until all possible orientations are measured. In a last step, the reference is measured again to take into account changes due to environmental influences, such as a slightly changed cavity temperature.

All measurements were taken using a Rhode & Schwarz ZVL network analyzer at an IF Bandwidth of 1 kHz and 601 points. The data are automatically collected in one-second intervals using a Matlab script. To ensure a high measurement accuracy, the center frequency of the VNA is set to the resonance frequency of the cavity at the beginning of each measurement cycle. The span is set to four times the measured 3 dB bandwidth.

## 3. Results

**Figure 7:**
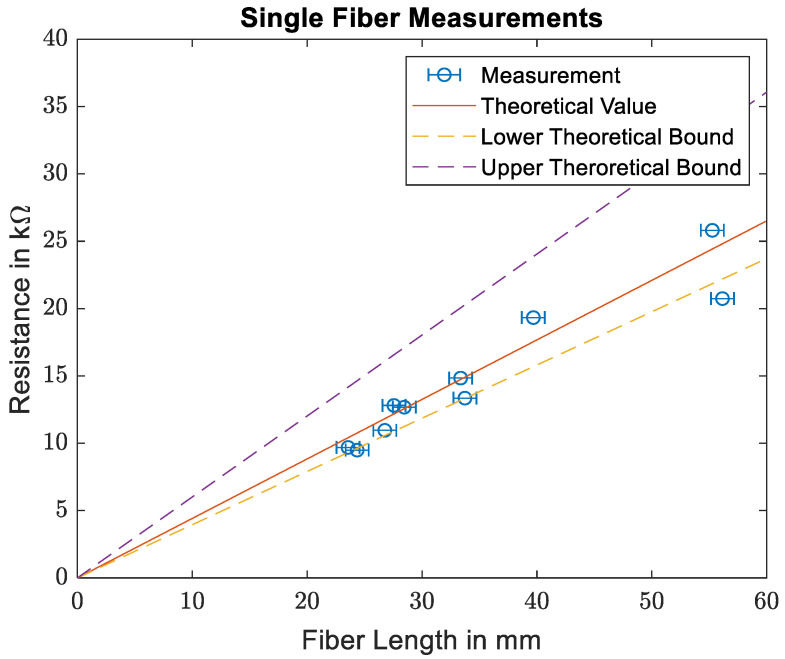
Comparison of the measured resistances for single carbon fibers with the data provided in the data sheet.

Figure 7 shows the comparison of the measured resistivities for the single carbon fibers and the theoretical values based on the manufacturer’s information. As the measured resistances are in the kΩ range, the contact resistances between the carbon fibers and the copper electrodes as well as between the copper electrodes and the multimeter electrodes are neglected. While the data sheet for the T300 carbon fibers specifies a fiber diameter of 7 µm, measurements of fiber diameters presented in [10] show that the actual diameter varies between 6 µm and 7.4 µm. The solid line denotes theoretical resistance based on the fiber diameter given in the data sheet, while the dashed lines denote the boundaries of the theoretical resistance based on the measured fiber diameters in [10]. As the fibers are not always placed perfectly straight, an error of up to 1 mm is assumed for the fiber length measurement. As can be seen, the theoretic and measured values coincide quite well. Going forward, the fiber conductivity of $5.88\cdot 10^{5}S/m$ specified by the manufacturer is used.

The measured quality factors of the prepreg samples are presented in Table 1. In contrast to the results for the aluminum lid shown above, the measurements of the prepreg samples show some variation between the different sample orientations. This is attributed to differences in sample placement due to the lower precision of the hand-cut prepreg sample’s dimensions compared to the machined aluminum lid. While Samples 1 and 2 show very similar results, the results of Sample 3 deviate slightly from the other two. This can be explained as Sample 3 having warped slightly during curing, which prevents it from laying perfectly flat in the sample holder.

The value of the Q factor for the aluminum reference is measured before and after all prepreg measurements and varies between 4847 and 4872. After generating matching characteristic curves with CST Microwave Studio, the lower and upper limits of the conductivities of the three samples can be determined. These values are also presented in Table 1.

For the measurements of the CFRTP samples, the measured Q of the aluminum reference varies between 4849 and 4861. The results for the CFRTP samples are presented in Table 2. Like the prepreg samples, the CFRTP samples show a clear influence on sample placement. However, in contrast to the prepreg samples, the influence of the sample surface is much higher compared to other repositioning influences. This is especially apparent for Sample 6, for which one side has a significant amount of surface defects which are visible with the naked eye. One of these defects can be seen in Figure 6f in the bottom right corner. An optical profilometry measurement of Sample 6 provided a root mean square surface roughness of 24 µm for the surface with defects and 2.5 µm for the smooth surface on the opposite side. For the other samples, there is no apparent quality difference between the surfaces. This fits the measurement results in Table 2, as Sample 6 has a significantly larger range between the minimum and maximum of the measured Q than other samples.

**Figure 8:**
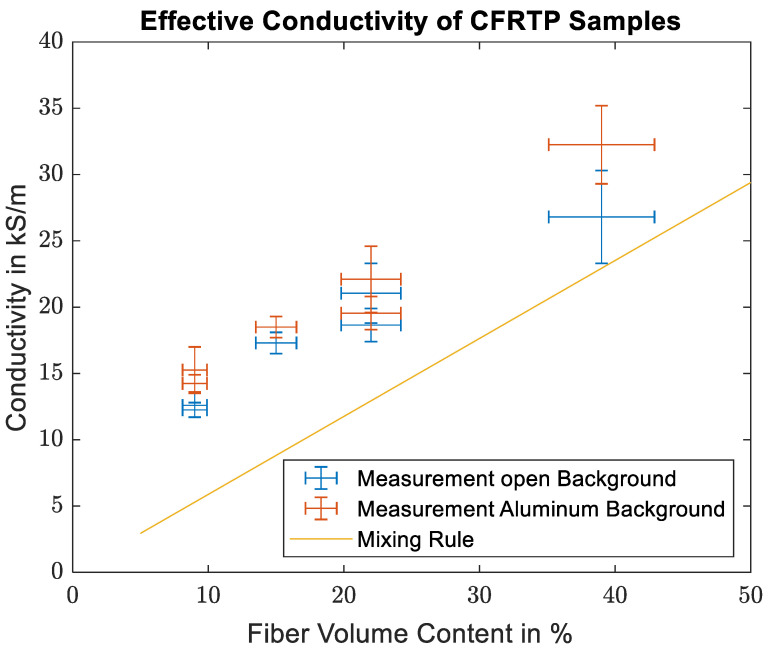
Comparison of the calculated effective conductivities of the CFRTP samples with the theoretical values found using the mixing rule.

The effective conductivities are then calculated in the same way as for the prepreg samples. Figure 8 shows a comparison between the effective conductivities measured for the thermoplastic samples, the effective conductivity calculated from Equation (3b), and the specified fiber conductivity. As predicted by the mixing rule, the measured conductivities show a linear dependence on the fiber volume content. There is, however, a significant offset between the measured and the theoretical values.

## 4. Discussion

The measurement results for the aluminum lid show that the system allows highly repeatable measurements if the sample is precisely fit to the sample holder. As the CFRP samples were produced by hand and are less rigid than the aluminum reference, there is a higher variation between the individual measurements due to positioning errors. The measurement of the prepreg samples show much better reproducibility than the handmade thermoplastic samples. This is mainly due to the industrial production process, which produces very homogeneous samples with even fiber distribution and straight fibers. In addition, the prepreg samples could be made thicker, resulting in higher rigidity and lower influence of the aluminum lid. While great care has been taken during the assembly of the handmade samples, the nature of the individual filaments bonded together leads to a more uneven fiber distribution. In addition, the fibers can shift when the matrix is melted, which reduces the parallelity of the fibers. However, the individual orientations of the handmade samples also show good measurement reproducibility.

As mentioned above, while the conductivities from the CFRTP measurements show the linear trend between the number of conducting filaments and the measured conductivity as expected, the exact values show a distinct offset to the mixing rule. This difference is caused by the imperfect distribution of the fibers in the sample compared to the homogeneous distribution assumed in the mixing rule. From the fiber conductivity given in the datasheet, the skin depth within the carbon fibers is 42 µm. While this is larger than the single fiber diameter of 7 µm, it is still much smaller than the diameters of the fiber bundles. Therefore, mainly the first few layers of fiber in each bundle contribute to the measured surface impedance. Bundles in the second row or unreinforced plastic in front or between the bundles do not significantly influence the surface impedance. A more accurate way of describing the sample should therefore take into account the sample surface covered by the fiber bundles. Judging from the detail picture in Figure 6, the fiber distribution within the bundle can be considered homogeneous. Therefore, the classical mixing rule can be used to calculate the effective conductivity of the fiber bundle. The fiber volume content within the bundle was determined from the SEM images to be between 60% and 65%. The effective conductivity of the sample is then estimated from the product of the effective conductivity of the bundle and the bundle covered sample surface. The covered surface was estimated from the microscope images in Figure 5b, and an estimate for the error was made from the variations in the individual segments from the detailed images of Sample 1 shown in Figure 5a. The maximum deviation of the segments to the total covered area fraction is 21.1%.

**Figure 9:**
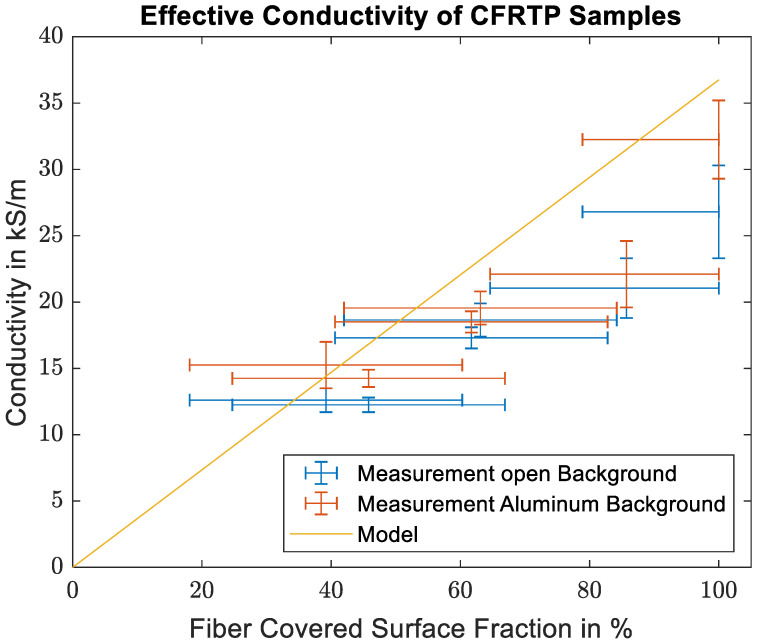
Comparison of the calculated effective conductivities of the CFRTP samples with the modified model using the surface covered by fiber bundles and the effective conductivity of fiber bundles.

Figure 9 shows the comparison of the measured values with the estimated values using the fiber covered surface. While the agreement of the measured and estimated data points is not perfect, the approach using the covered surface gives a reasonable estimate for the effective conductivity.

**Figure 10:**
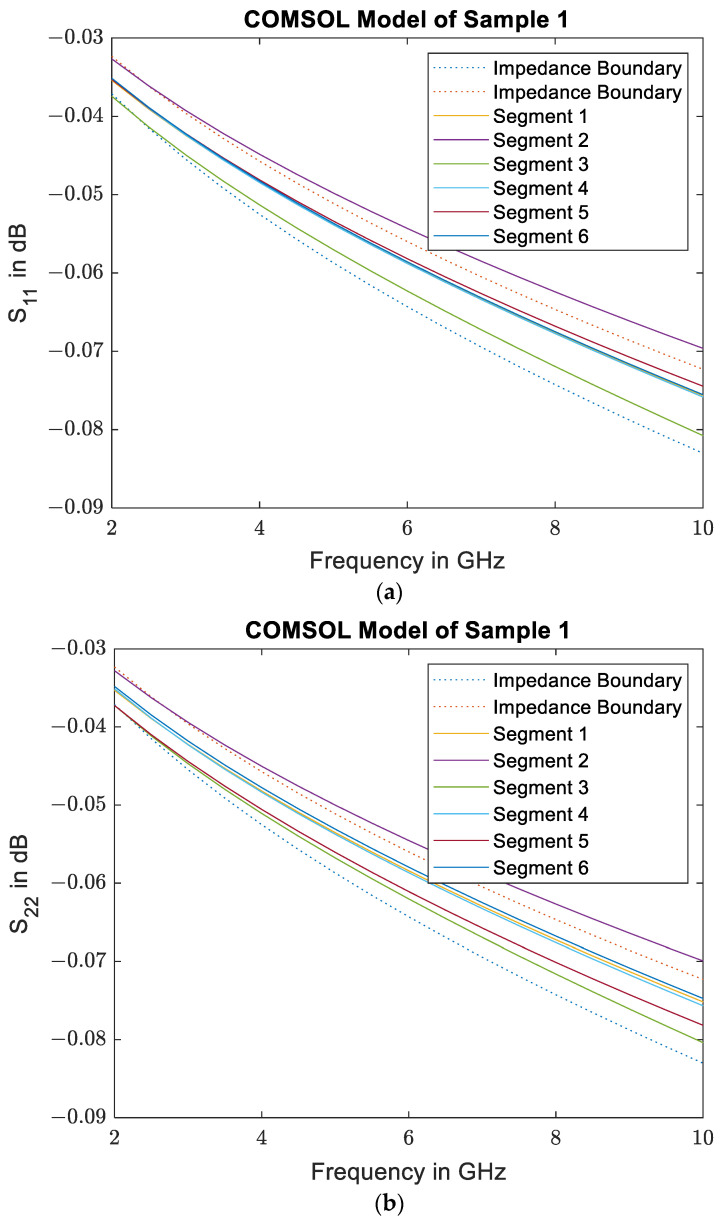
Results from the 2D COMSOL Model: S_11_ (**a**) and S_22_ (**b**) of a plane wave incident on the segments of Sample 1 shown in Figure 5 (solid lines) compared to S_11_ of ideal impedance boundaries (dotted lines). The frequency dependence of the sample deviates from the ideal boundary.

To obtain an understanding of the local variation of the effective conductivity due to the irregular placement of the fiber bundles, a planar 2D COMSOL model is set up from the detailed images of sample 1. Each of the sample segments is simulated individually. The fiber bundles are modeled using solid ellipses with the effective conductivity calculated from the Maxwell–Garnett rule. The upper and lower boundaries are set to be perfect magnetic conductors, which results in a plane wave that is incident on the sample with the electric field and induced currents oriented alongside the fibers in the out-of-plane direction. The S-parameters resulting from the simulation are depicted in Figure 10, and the validity of the very small S_11_ values was checked using a mesh refinement study. From looking at the reflection of the wave at both sample surfaces, it is obvious that there is some variation between the different segments. In addition to the shift in the curves, there is also some variation in the frequency dependence of the individual segments. This implies a frequency-dependent influence of the exact geometries on the material’s effective conductivity. A mixing rule can therefore only provide a rough estimate of the effective conductivity of such an irregular sample. Further examination of the reflection shows a significantly higher phase shift than expected for an impedance boundary with comparable conductivity. The effective conductivity of the sample segments can therefore not be calculated from the simulated reflection coefficient. The absolute value of the simulated reflection coefficient can, however, be compared to the reflection coefficient of an ideal impedance boundary. The reflection of the impedance boundaries is also included in Figure 10. Due to the variance in frequency dependence the impedance boundaries can only be matched at a single frequency. In this case, the boundary was matched at 2.45 GHz, as it corresponds to the operating frequency of the measurement system. The conductivities found for the impedance boundaries were 12.2 kS/m and 16.1 kS/m, which coincides well with the measured bounds for Sample 1, which are 11.7 kS/m and 14.9 kS/m.

## 5. Conclusions

The measurement system presented in this work allows easy measurements of the effective conductivity of flat samples by simply laying them on top of the cavity. Due to the shape of the characteristic curve, the system is especially suited for measurements of samples with conductivities between $10^{3}$ S/m and $10^{5}$ S/m. The cavity was designed to achieve unidirectional surface currents in the sample, which allows accurate measurements of axially anisotropic materials such as carbon fiber composites. For the samples presented here, the measurement accuracy is mainly limited by the sample homogeneity and surface quality, as multiple measurements of the same sample in the same orientation show very good agreement. While there are some inconsistencies between the classical mixing rules and measured values of the CFRTP samples, this can be explained by the irregular sample geometry caused by the manual sample preparation process. A detailed simulation model based on the true sample geometry resulted in effective conductivity values that match the measured values closely.

## Figures and Tables

**Table 1:** Minimum and maximum measured Q values for the prepreg samples with the corresponding effective conductivities.

Sample:	Min Q	Max Q	Min Conductivity in kS/m	Max Conductivity in kS/m
Prepreg Sample 1	3723	3792	29.8	36
Prepreg Sample 2	3739	3796	30.6	36.4
Prepreg Sample 3	3685	3776	27.4	34.7

**Table 2:** Minimum and maximum measured Q values for the CFRTP samples with different fiber volume contents.

Sample:	Fiber Volume Content	Minimum Q	Maximum Q
CFRTP Sample 1	9%	3250	3372
CFRTP Sample 2	9%	3246	3440
CFRTP Sample 3	15%	3432	3505
CFRTP Sample 4	22%	3458	3543
CFRTP Sample 5	22%	3499	3624
CFRTP Sample 6	39%	3602	3783
